# Sea cucumber protein hydrolysate restores the Th1/Th2 paradigm in cyclophosphamide-induced immunosuppressed mice

**DOI:** 10.3389/fnut.2026.1758733

**Published:** 2026-01-27

**Authors:** Nabeel Ahmed Farooqui, Ata Ur Rehman, Asif Iqbal Khan, Hidayat Ullah, Muhsin Ali, Waleed Yousuf, Yamina Alioui, Aamna Atta, Mohammad Abusidu, Bilal Saleh, Eslam Ghaleb, Yanxia Li, Yi Xin, Nimra Zafar Siddiqui, Liang Wang

**Affiliations:** 1 Department of Biotechnology, College of Basic Medical Science, Dalian Medical University, Dalian, China; 2 Department of Anesthesiology, Duke University Medical Center, Durham, NC, United States; 3 Dow Institute of Medical Technology, Dow University of Health Sciences, Karachi, Pakistan; 4 Guangdong Provincial Key Laboratory of Research and Development of Natural Drugs, and School of Pharmacy, Guangdong Medical University, Dongguan, China; 5 Institute of Cancer Stem Cell, Dalian Medical University, Dalian, China; 6 Multidisciplinary Research Lab, Bahria University of Health Sciences, Karachi, Pakistan; 7 Department of Respiratory and Clinical Care Medicine, Institute of Respiratory Diseases, The First Affiliated Hospital of Dalian Medical University, Dalian, China; 8 Stem Cell Clinical Research Center, National Joint Engineering Laboratory, Regenerative Medicine Center, The First Affiliated Hospital at Dalian Medical University, Dalian, China

**Keywords:** *Apostichopus japonicus*, collagenase-I, immunocompetence, immuno-nutraceutical, immunosuppression, sea cucumber protein hydrolysate, Th1/Th2 homeostasis

## Abstract

**Introduction:**

Immunocompetence reflects the immune system’s capacity to mount effective responses against antigens. Cyclophosphamide (CYP), a chemotherapeutic and experimental immunosuppressive agent, disrupts T-helper (Th)1/Th2 balance and compromises immune organ integrity. Bioactive peptides derived from the sea cucumber *Apostichopus japonicus*, which is rich in type I collagen, have shown emerging immunomodulatory potential.

**Methods:**

A collagenase-derived sea cucumber protein hydrolysate (SCPH) was prepared using Clostridium collagenase-I and characterized for amino acid composition and peptide profiles. BALB/c mice were administered CYP (80 mg/kg, i.p.) to induce immunosuppression, followed by oral SCPH treatment. Body weight, spleen and thymus indices, leukocyte counts, Th1/Th2-associated gene expression (IFNG, TBX21, IL4, GATA3), serum cytokines and immunoglobulins, and splenic histology and immunohistochemistry were evaluated.

**Results:**

CYP treatment induced body weight loss, reduced immune organ indices, altered leukocyte profiles, and disrupted Th1/Th2-associated markers. SCPH administration partially reversed these changes by restoring spleen and thymus indices, normalizing circulating immune cell levels, upregulating Th1-associated genes (IFNG, TBX21), and downregulating Th2-associated genes (IL4, GATA3). SCPH also increased serum Th1 mediators (IFNG, IgG, IgM) while reducing Th2-associated markers (IL-4, IL-10, IgA, sIgA). Histological and immunohistochemical analyses confirmed improved splenic architecture with elevated T-bet and reduced GATA3 expression.

**Discussion/conclusion:**

These findings indicate that SCPH promotes Th1-biased immune rebalancing in CYP-induced immunosuppressed mice, suggesting its potential as a marine-derived immunonutritional agent. Further mechanistic studies are warranted to explore its therapeutic and translational applications.

## 1 Introduction

Our immune system is a complex framework comprising cells, tissues, and organs primarily involved and work in conjunction for the body’s defense mechanisms. Under normal conditions, it discriminates effectively between self and non-self, mounting immune responses against harmful agents while sparing healthy tissues. Contrary to this, when the immune system erroneously detects healthy tissues or substances as unhealthy, it activates an unnecessary immune response, resulting in autoimmune diseases and allergies (1).Immunocompetence concerns to the proficiency of the mature immune system (primarily B and T lymphocytes) to mount effective responses against antigens such as toxins, pathogens, and damaged cells, thereby maintaining inflammatory homeostasis (2, 3). This competence depends on the functional integrity of macrophages and lymphocytes, as well as the hormonal regulation of immune responsiveness (3). A further key component is the adaptive memory of B and T cells, which enables rapid and robust responses to previously encountered antigens (4). Immunocompetence not only supports effective host defense but also restrains excessive or misdirected immune responses, thereby limiting autoimmunity and allergy and preserving immune homeostasis. At the organ level, distinct lymphoid and non-lymphoid tissues contribute specialized roles in immune cell production, maturation, and activation (5, 6).

T helper (Th) cells are central to immune regulation, and imbalance between Th1 and Th2 subsets is a hallmark of dysregulated immunity. Excessive Th2 activity can suppress Th1 function and promote immunosuppressive states. Th1 cells drive cell-mediated immunity against intracellular pathogens, malignant cells, and mediate delayed-type hypersensitivity reactions, whereas Th2 cells support antibody-mediated responses against extracellular pathogens (7, 8). The transcription factor T-bet (encoded by *TBX21*) is the master regulator of Th1 differentiation; its activation upregulates hallmark Th1 cytokines, including IFNG and IL1B, while repressing Th2-associated cytokines such as IL4, IL10, and IL13. T-bet also inhibits GATA3 function through direct interaction or binding to GATA3 regulatory regions, establishing a cross-inhibitory axis in which Th1 differentiation suppresses the alternative Th2 pathway (9–11). Conversely, GATA3 is the key regulator of Th2 differentiation, promoting expression of IL4, IL5, IL10, and IL13 and repressing Th1-related cytokines, including IFNG and IL1B, thereby skewing responses toward a Th2 phenotype (12, 13). In immunosuppressive states, perturbation of Th1/Th2 homeostasis, characterized by increased Th2 activity and diminished Th1 responses, contributes to an overall immunosuppressive microenvironment (7, 8).

Collagen is the principal fibrillar protein in most living organisms, and type I collagen is the predominant isoform in the tissues of many marine invertebrates. In sea cucumbers, collagen can comprise up to 70% of the body wall, and its hydrolysis yields low-molecular-weight peptides with diverse biological activities, including anticancer, antihypertensive, immunomodulatory, neuroprotective, and cartilage-repair effects (14, 15). The bioactivity of sea cucumber collagen hydrolysates reflects their unique peptide sequences and composition. Sea cucumber–derived collagen is notable for its relatively high yield and distinctive amino acid profile, enriched in glycine, proline, and hydroxyproline, distinguishing it from other collagen sources. Additional functional diversity arises from species-specific differences and the extraction and hydrolysis conditions used for peptide production (14, 15). Discovered in 1953, the first marine-derived bacterial (*Clostridium histolyticum*) collagenase has been recognized for its ability to cleave the triple-helical collagen structure (resistant to other proteases), even in complex body tissue under mild processing conditions. Thereby, conserving the nutritional and bioactive properties of the collagenous peptides in the hydrolysate (16, 17).

To our knowledge, no study has used *Clostridium* collagenase-I to generate an *A. japonicus* protein hydrolysate and systematically examine Th1/Th2-related gene expression (*IFNG*, *TBX21*, *IL4,* and *GATA3*), cytokines, immunoglobulins, and T-bet/GATA3 protein distribution in a CYP-induced immunosuppressed mice model.

## 2 Methodology

### 2.1 Chemicals and reagents

Sea cucumbers were acquired from the sea seafood market of Lvshunkou, Dalian, China. Collagenase-I (extracted from *Clostridium histolyticum*) was purchased from Biosharp Life Sciences (China). CYP and chloroform were purchased from Sigma-Aldrich (USA). EDTA tubes were bought from Shijiazhuang Medical Company (China). 4% Paraformaldehyde from Servicebio (China), Levamisole hydrochloride (LH), and nuclease-free water were bought from Solarbio (China). ELISA assay kits for IL4, IL10, IFNG, IL1B, IgM, IgG, IgA and sIgA were obtained from JONLNBIO (China). TRIzol reagent from ThermoFisher Scientific (USA), ethanol, and isopropanol were supplied by Shanghai Sinopharm Reagent Group (China). HiScript III RT SuperMix for cDNA preparation and ChamQ Universal SYBR qPCR Master Mix for qPCR were acquired from Vazyme Biotech (China). Gene primers for *IL4*, *IFNG*, *TBX21*, *GATA3*, and *ACTNB* were sourced from Sangon Biotech (China). T-bet and GATA3 primary antibodies were sourced from Protein-tech (China). IHC kit and DAB were purchased from MXB biotechnologies (China). All the other reagents were of analytical grade.

### 2.2 Preparation of SCPH from *Apostichopus japonicus*

**Figure 1:**
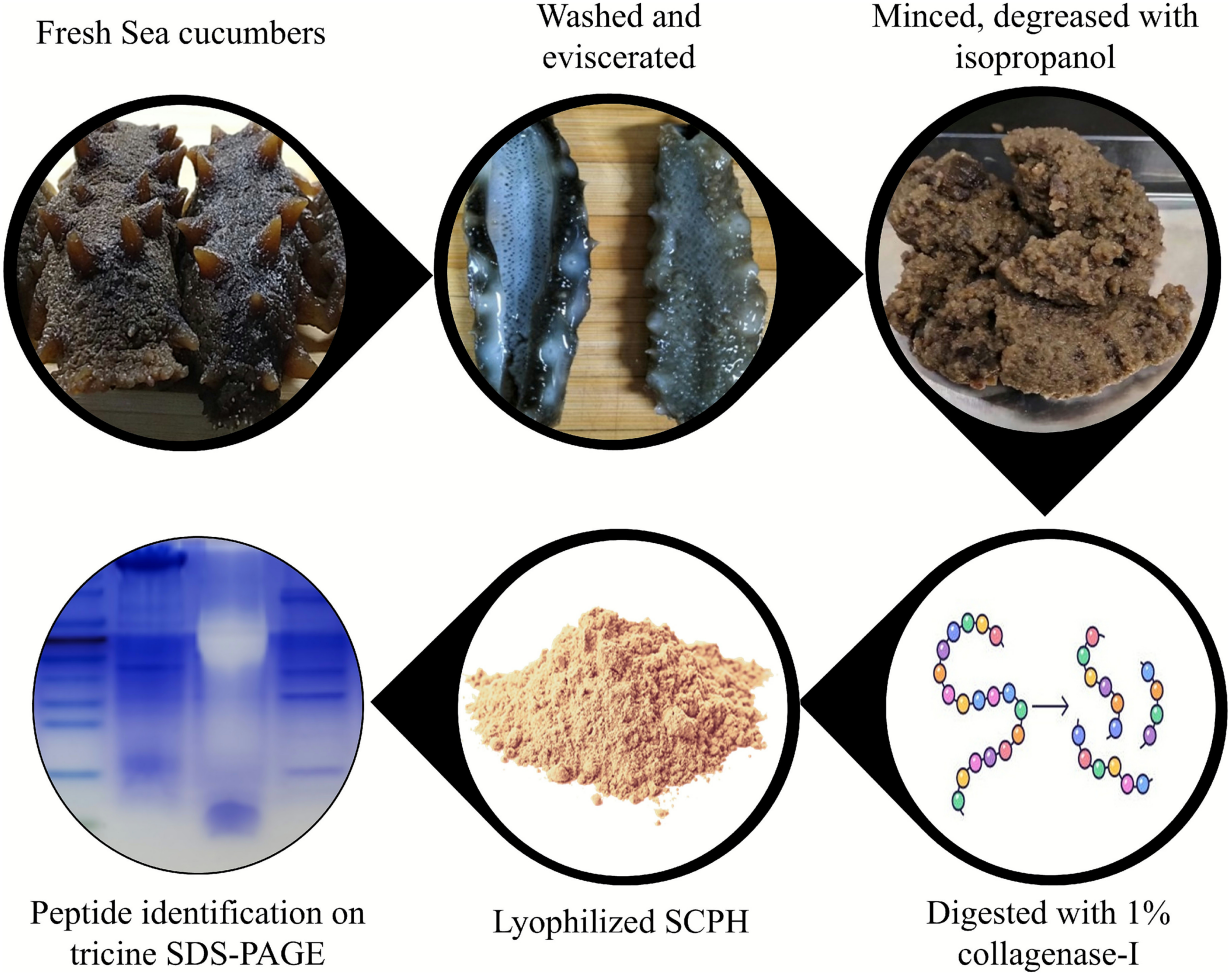
Compendious framework of SCPH preparation and identification after enzymatic hydrolysis.

The entire sea cucumbers (*A. japonicus*) were eviscerated, minced, and homogenized in ice-cold distilled water (DW) with mild sonication (2 min on/3 min rest cycles) for 30 min, followed by defatting with isopropanol (1,4, w/v) under constant stirring for 45 min. The suspension was centrifuged at 3,000 rpm for 20 min, and the resulting sludge was lyophilized and stored at −80 °C as CPL. For enzymatic hydrolysis, 1 g of lyophilized CPL was resuspended in 80 mL PBS, supplemented with 0.5 mM CaCl₂ and 1% (w/w) collagenase I, and incubated at 40 °C with shaking at 120 rpm for 0–5 h. The reaction was heat-inactivated by boiling for 10 min at each time point, and the mixture was centrifuged at 14,000 rpm for 10 min to collect low-molecular-weight peptides in the supernatant. SCPH yield was quantified by Bradford assay, with maximal peptide yield observed at 5 h; this procedure was repeated three times to ensure batch-to-batch consistency. The 5 h supernatant was concentrated to 20 mL by rotary evaporation at 40 °C, freeze-dried to obtain SCPH powder, and stored at −80 °C (Figure 1).

The molecular weight distribution of SCPH peptides was analyzed by Tris–tricine SDS–PAGE for high-resolution separation of low-molecular-weight peptides. Dried SCPH powder was dissolved in DW, and 50 μg of peptide per lane was loaded onto a Tris–tricine gel consisting of a 4% stacking gel, a 10% upper resolving gel, and a 15% lower resolving gel. Electrophoresis was carried out at 100 V until complete separation was achieved. Gels were stained for 30 min, rinsed with DW, and destained overnight, after which peptide bands were visualized using a light transilluminator.

### 2.3 Amino acid analysis and molecular mass identification of SCPH

Amino acid composition and abundance in SCPH were determined using an LA8080 high-speed amino acid analyzer (Hitachi, Japan) employing ninhydrin-based ion-exchange chromatography. Samples (20 μL) were injected onto a cation-exchange column (3 μm), eluted isocratically with sodium citrate buffer at 0.4 mL/min, and post-column derivatized with ninhydrin; derivatives were detected at 570 nm and 440 nm to quantify primary and amino acids, respectively, at a column temperature of 57 °C. Peptide molecular masses in SCPH were analyzed by liquid chromatography–tandem mass spectrometry (LC–MS/MS). SCPH was diluted in 0.2% formic acid and loaded onto a C18 reversed-phase column equilibrated with eluent A (water + 0.1% formic acid) and eluent B (acetonitrile + 0.1% formic acid) at a constant flow rate of 5 μL/min, with the column maintained at 30 °C and a total acquisition time of 0–75 min. Peptide retention time, mass-to-charge ratio (m/z), precursor molecular weight, X-correlation score, and charge state were obtained from LC–MS/MS data for peptide characterization. Sequence similarity, alignment scores, and E-values of identified peptides were evaluated by BLASTp against the non-redundant protein database to confirm peptide identity and infer putative protein origins.

### 2.4 Animal housing and experimental design

Adult female BALB/c mice (4–5 weeks, 18–22 g; *n* = 36) were acclimatized for 7 days under standard conditions (12 h light/dark, 25 ± 2 °C, 55 ± 10% humidity). After ethical approval (Dalian Medical University, 202,310,261), mice were divided into 6 groups (*n* = 6/group): normal control (NC; saline gavage), CYP (80 mg/kg i.p. × 4 days then saline), levamisole (LH; 40 mg/kg), non-hydrolyzed protein lysate (CPL; 300 mg/kg), and SCPH low/high dose (LD/HD; 100/300 mg/kg). The dose of SCPH was selected based on its enrichment in low-molecular-weight peptides generated by collagenase digestion, which confers higher peptide density and bioavailability compared with the crude protein fraction (CPL). Therefore, SCPH was administered at 100 mg/kg (low dose) and 300 mg/kg (high dose) to evaluate dose-dependent effects. CPL, consisting of non-hydrolyzed intact proteins, was administered at 300 mg/kg to approximate the total protein input and potential peptide equivalents provided by the low dose of SCPH (100 mg/kg). CYP dosing followed established immunosuppression models (18), showing reduced organ indices and leukocyte counts. SCPH doses were selected based on marine peptide literature (50–500 mg/kg) and pilot data requiring ≥100 mg/kg for immune effects.

**Figure 2:**
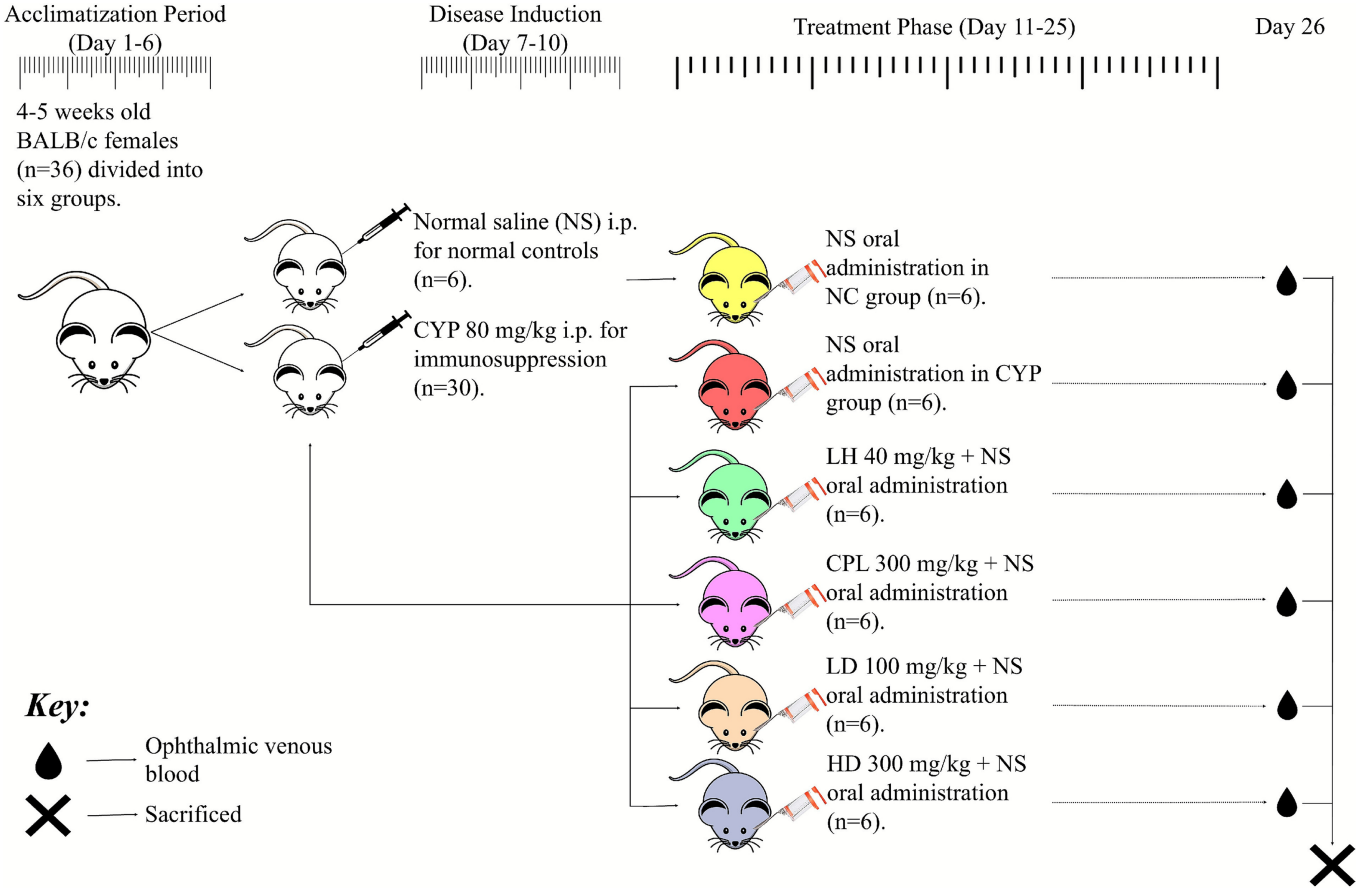
Experimental timeline of acclimatization, immunosuppression, and SCPH supplementation phase in BALB/c mice models.

The body weight of each group was noted daily throughout the experiment. The experimental timeline is detailed in Figure 2. After the successful construction of immunosuppressed mouse models, the final body weight of mice was recorded on the 26th day, and blood was collected for immune cell counts after being sacrificed. The whole organs, i.e., spleen, thymus, and mesenteric lymph nodes (MLN), were taken for organ indexing, and spleen tissues were fixed in 4% paraformaldehyde solution and then sectioned for hematoxylin and eosin (HE) staining and immunohistochemistry (IHC) experiments.

### 2.5 Evaluation of immune organ indices and immunocytes

The mice spleen, thymus, and MLN were exenterated and cleaned in sterile-chilled PBS, gently dried on sterile filter paper, and weighed. The weighted organ (g) / total mice weight (g) × 100 was used to measure the indices for each group. Blood was collected in EDTA tubes of at least 200 μL and put in a 4 °C refrigerator for a week, and then sent to Dalian Medical University hospital for analysis of immune cells. An automatic animal blood cell analyzer (BC-2800 Vet) was used to evaluate white blood cell, lymphocyte, monocyte, and granulocyte counts and their relative percentages.

### 2.6 Tissue RNA isolation

RNA was extracted from spleen tissue to quantify the expression of Th1 and Th2-associated genes by qPCR, including the Th1-associated transcription factor *TBX21* and cytokine *IFNG*, and the Th2-associated transcription factor *GATA3* and cytokine *IL4*. Total RNA was isolated using the TRIzol method. Briefly, 100 mg of spleen (rinsed in sterile PBS) was placed in a 2 mL conical tube, supplemented with 1 mL chilled TRIzol reagent, and homogenized. The homogenate was centrifuged at 12,000 × g for 10 min, and the clear supernatant was transferred to a fresh tube, avoiding tissue debris. Chloroform (0.2 mL per 1 mL TRIzol) was added, the tube was gently inverted several times, incubated at room temperature for 10 min, and centrifuged for 10 min to separate phases. The upper aqueous phase (400–500 μL) was collected carefully without disturbing the interphase, mixed with an equal volume of isopropanol, incubated for 10 min at room temperature, and centrifuged at 12,000 × g for 10 min at 4 °C to pellet RNA. The supernatant was discarded, and the pellet was washed twice with 75% ethanol (prepared in nuclease-free water) by centrifugation at 7,500 × g for 5 min at 4 °C, then air-dried and resuspended in 20 μL nuclease-free water. RNA concentration and purity were assessed using a NanoDrop 2000 spectrophotometer (Thermo Fisher Scientific) by measuring absorbance at 260 nm and 280 nm.

### 2.7 cDNA preparation and reverse transcription-quantitative polymerase chain reaction (RT-qPCR)

1 μL of RNA (concentration adjusted to 1,000 ng) was taken with 4 μL gDNA wiper into 7 μL nuclease-free water in an RNase-free PCR tube, mixed thoroughly with positive displacement, and incubated at 42 °C for 2 min for genomic DNA removal, and the tubes were pulse centrifuged to settle down the components. 4 μL 5 × HiScript III qRT SuperMix, then mix the components with the aid of positive displacement and incubate at 42 °C for 15 min for reverse transcription and 85 °C for 5 s for reaction termination. Pulse centrifuges the tubes, followed by reverse transcription; freshly prepared cDNA was then diluted to four times and mixed thoroughly by positive displacement. In the new RNase-free PCR tubes, add 0.4 μL forward, 0.4 μL reverse (10 μM each) primers, 8.2 μL nuclease-free water, 1 μL template, and light-sensitive 10 μL ChamQ Master Mix (2×) to make the 20 μL reaction volume. The thermocycling conditions were set as: pre-denaturation at 95 °C for 30 s, amplification at 95 °C for 10 s, and 60 °C for 30 s up to 40 cycles, extended to dissociation melt. The triplicates of four samples were amplified from each group to evaluate the relative fold change, and the *ACTNB* was utilized as an internal calibrator with each target gene. The set of validated primer sequences is mentioned in Table 1.

**Table 1:** List of primers used from the RT-qPCR experiment.

Target	Sequence (5′ – 3′)
*TBX21*	F: CGTTTCTACCCCGACCTTCC R: ATGCTCACAGCTCGGAACTC
*IFNG*	F: CAGCAACAGCAAGGCGAAAAAGG R: TTTCCGCTTCCTGAGGCTGGAT
*GATA3*	F: AAGCTCAGTATCCGCTGACG R: GATACCTCTGCACCGTAGCC
*IL4*	F: ACCAGGAGCCATATCCACGGATG R: GGTGTTCTTCGTTGCTGTGAGGAC
*ACTNB*	F: ATCGCTGCGCTGGTCG R: GTCCTTCTGACCCATTCCCA

### 2.8 Immunoassay of serum Th1 and Th2 cytokines and immunoglobulins

Sandwich enzyme-linked immunosorbent assays (ELISAs) were used to quantify Th1- and Th2-associated cytokines (IL4, IL10, IFNG, IL1B), and competitive ELISAs were used to measure immunoglobulins (IgG, IgM, IgA, sIgA) in serum and intestinal lumen samples following SCPH treatment. Whole blood was centrifuged at 1500 × g for 20 min to obtain serum, and intestinal contents were incubated overnight at 4 °C and then centrifuged at 1,000 × g for 20 min to collect supernatants for sIgA measurement; all samples were diluted 1:1,000 by adding 50 μL sample to 950 μL universal diluent and mixing thoroughly. For cytokine sandwich ELISAs, plates and reagents were equilibrated to room temperature for 15 min, then 100 μL of serially diluted standards and 100 μL of diluted samples were added to designated wells, the plate was sealed, and incubated at 37 °C for 60 min. After aspirating contents, 100 μL of 1 × biotinylated detection antibody was added to each well, the plate was resealed and incubated, and wells were subsequently washed three times with 300 μL of 1 × wash buffer (prepared from a 20 × stock), with 1 min soaking per wash followed by aspiration on absorbent paper. Streptavidin–horseradish peroxidase (HRP; 1×, 100 μL per well) was then added, incubated at 37 °C for 30 min, and removed, followed by five wash cycles as above. Tetramethylbenzidine (TMB) substrate (90 μL per well) was added, and plates were incubated at 37 °C for 15 min in the dark, after which 50 μL stop solution was added to each well and absorbance was read immediately at 450 nm on a microplate reader.

Competitive ELISA utilized to quantify immunoglobulins, 50 μL of standards or diluted samples and 50 μL universal diluent were added to the appropriate wells, followed by 50 μL HRP-conjugated antigen, and plates were sealed and incubated at 37 °C for 60 min. Wells were then washed five times with 300 μL of diluted wash buffer (1 min soak per wash and aspiration between washes), followed by addition of 90 μL TMB substrate, plate sealing, and incubation at 37 °C for 15 min. The reaction was terminated by adding 50 μL stop solution per well, and absorbance was measured immediately at 450 nm using a microplate reader. All assays were performed in duplicate, and four biological samples per group were analyzed to quantify cytokine and immunoglobulin levels in crude serum and intestinal samples.

### 2.9 Tissue sectioning and histopathological evaluation of spleen tissues

Spleens (~200 mg; *n* = 3 per group) were fixed in 4% paraformaldehyde for 24 h, embedded in paraffin, and sectioned at 5 μm thickness using a microtome. HE staining was performed following dehydration in an ethanol series (100, 95, 70, 50%) and xylene clearing. Sections were rehydrated through reverse gradients (xylene, 100, 95, 70, 50% ethanol and DW), stained in hematoxylin for 6 min, briefly rinsed in tap water (10 s), differentiated in 1% acid alcohol (10 s), blued in weak alkaline alcohol (15 s), and counterstained in eosin for 1 min. After rinsing in tap water, sections were dehydrated in ascending ethanol gradients, cleared in xylene, and mounted in neutral balsam. Images were acquired at 10 × and 20 × magnification (100 μm scale bar) using Image-Pro Plus software.

### 2.10 Immunohistochemical analysis of T-bet and GATA3 protein expression

IHC was performed on 5-μm splenic sections from paraffin blocks to assess T-bet and GATA3 expression. Sections on glass slides were deparaffinized in xylene, rehydrated through descending ethanol gradients, and treated with 3% H₂O₂ for 10 min to quench endogenous peroxidase. Antigen retrieval was conducted in preheated citrate buffer (microwave, 15 min), followed by cooling to room temperature. Sections were incubated overnight at 4 °C with rabbit polyclonal anti-T-bet (1:500) or anti-GATA3 (1:500) primary antibodies, washed three times in PBS, and incubated with HRP-conjugated secondary antibody for 1 h at room temperature. After three 5-min PBS washes, DAB chromogen was applied for 1 min, followed by hematoxylin counterstaining. Sections were dehydrated, cleared in xylene, mounted with neutral balsam, and imaged at 10×, 20×, and 40 × magnification (100 μm scale bar) using a microscope. DAB intensity was quantified by densitometric analysis in ImageJ and normalized to hematoxylin-stained area.

### 2.11 Biostatistical analysis

IBM-SPSS (v25) was used for all data analysis and presented as the mean ± SD for each group. Normality was assessed by Shapiro–Wilk test. Normally distributed data used one-way ANOVA with Tukey’s post-hoc correction. Non-normal data used Kruskal-Wallis with pairwise comparisons among the groups. The accepted level of significance was *p* < 0.05. The graphs and charts were constructed with Microsoft Excel 365, SPSS (v25).

## 3 Results

### 3.1 Identification of peptides and amino acids in SCPH

**Figure 3:**
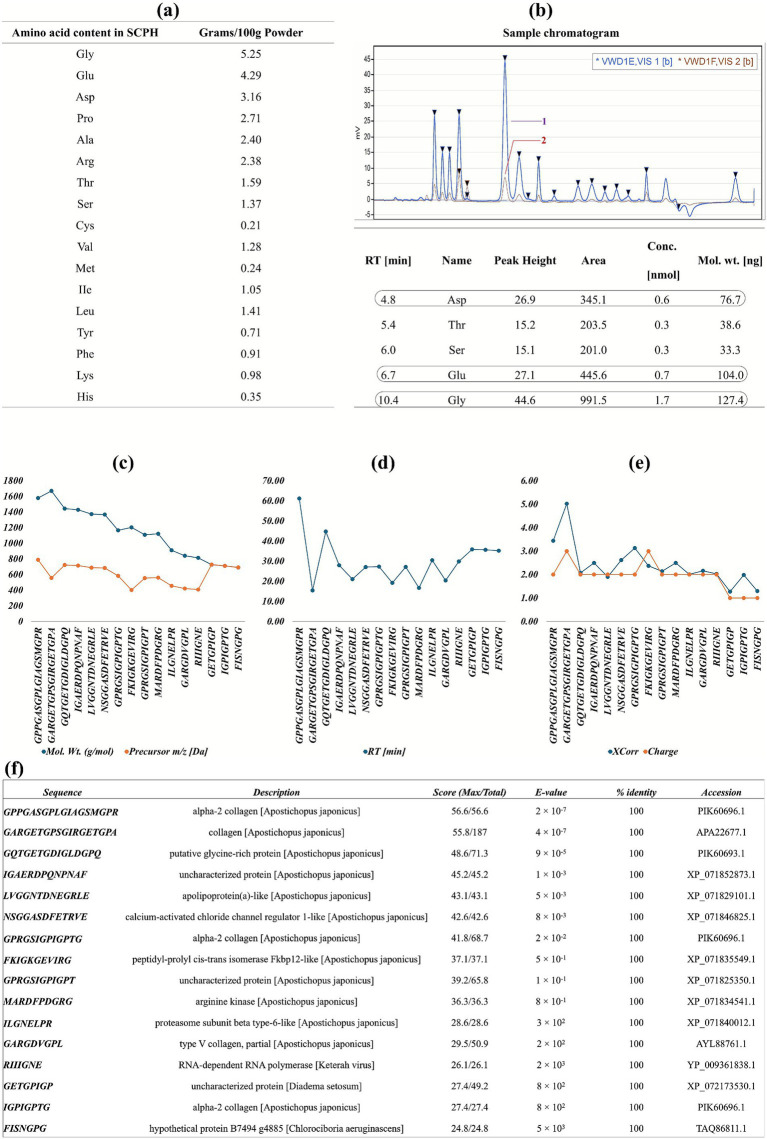
Amino acid content and its identification in SCPH. **(A)** Quantification of amino acids in the hydrolysate **(B)**. Peaks of amino acids on the ion-exchange chromatogram, with their molecular masses and retention time. Asp., Glu, and Gly have relatively higher peaks with maximum area coverage, and their molecular weights **(C)**. Molecular weight and charge to mass ratio **(D)**. Retention time **(E)**. X-correlation and charge of identified peptides **(F)**. Sequence alignment scores of the peptides in SCPH.

The PH 4-New HPLC method was used to detect the amino acids in the chromatogram. The amino acids Asp., Glu, and Gly are presented with higher coverage area (345.1, 445.6, and 991.5), peak height (26.9, 27.1, and 44.6), retention time (4.8, 6.7, and 10.4), and molecular weights (76.7, 104.0, and 127.4) in an Ion-Exchange chromatogram, respectively. VWDIE, VIS 1 (blue peak) is for effective separation, while VWDIF, VIS 2 (red peak) is essentially for basic amino acid analysis. The total amino acid content in the SCPH was found to be Gly, Glu, Asp., Pro, Ala, and Arg, which are abundantly present as 5.25, 4.29, 3.16, 2.71, 2.40, and 2.38 g per 100 g of SCPH. The peptide sequences were identified by LC-MS^2^ with their characteristics, i.e., retention time, charge to mass ratio, molecular weight, X-correlation, and charges illustrated in Figures 3A–E. The peptide identity, scores, and E-values were calculated by BLASTp and presented in Figure 3F.

### 3.2 SCPH enhances immune organ indices and elevates immunocyte counts

**Figure 4:**
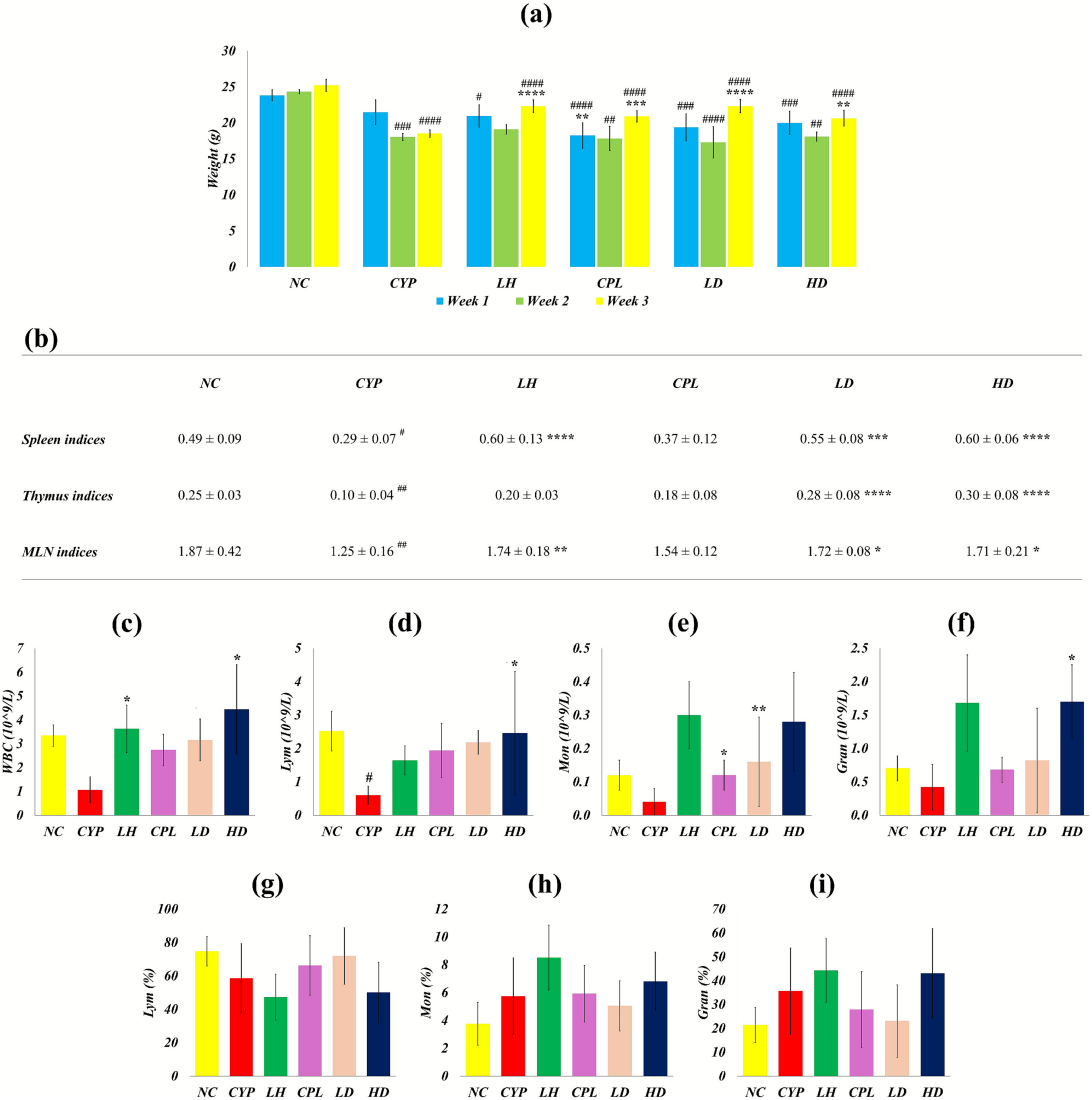
Body weight change, immune organ indices, and immunocyte counts and percentages after SCPH treatment. **(A)** Body weight changes from 1st to 3rd week **(B)** Rejuvenation of immune organs **(C)** Decreased cellular counts of **(D)** WBCs **(E)** Lymphocytes **(F)** Monocytes **(G)** Granulocytes and the percentages of **(H)** Lymphocytes, monocytes, and **(I)** Granulocytes found in the blood. Results are shown with Mean ± SD (*n* = 5). ^#^*p* < 0.05, ^##^*p* < 0.005, ^###^*p* < 0.0005, ^####^ *p* < 0.0001 compared with NC group; ^*^p < 0.05 ^**^ *p* < 0.005, ^***^p < 0.0005, ^****^p < 0.0001 compared with CYP group.

CYP reduced body weight and immune organ indices, consistent with systemic toxicity and targeted immunosuppression. Body weight in the CYP group declined relative to the normal control (NC) group during the first, second, and third weeks (*p* > 0.05, *p* = 0.005, and *p* > 0.0001, respectively), whereas SCPH-treated groups largely maintained body weight. Spleen, thymus, and mesenteric lymph node indices were significantly decreased in the CYP group compared with NC (*p* = 0.010, *p* = 0.002, and *p* = 0.001, respectively), and SCPH administration significantly promoted regeneration of these immune organs (Figures 4A,B), in line with reports that effective interventions can reverse CYP-induced loss of body and organ mass. CYP markedly altered hematopoietic dynamics, reflecting myelosuppression, while SCPH treatment attenuated these effects. Total WBC, lymphocyte, monocyte, and granulocyte counts were reduced in the CYP group relative to NC, reaching statistical significance only for lymphocytes (*p* > 0.05, *p* = 0.026, *p* > 0.05, and *p* > 0.05, respectively), a pattern characteristic of CYP-induced leukopenia and lymphopenia. SCPH treatment increased these cell counts, indicating partial restoration of bone marrow and peripheral immune function. In contrast to absolute counts, the proportions of lymphocytes, monocytes, and granulocytes showed non-significant elevations in the CYP group relative to NC, suggesting relative shifts in leukocyte composition driven by an overall fall in WBC number rather than true expansion of specific subsets (Figures 4C–I).

### 3.3 Association of SCPH with restoration of Th1/Th2 genes and cytokines

**Figure 5:**
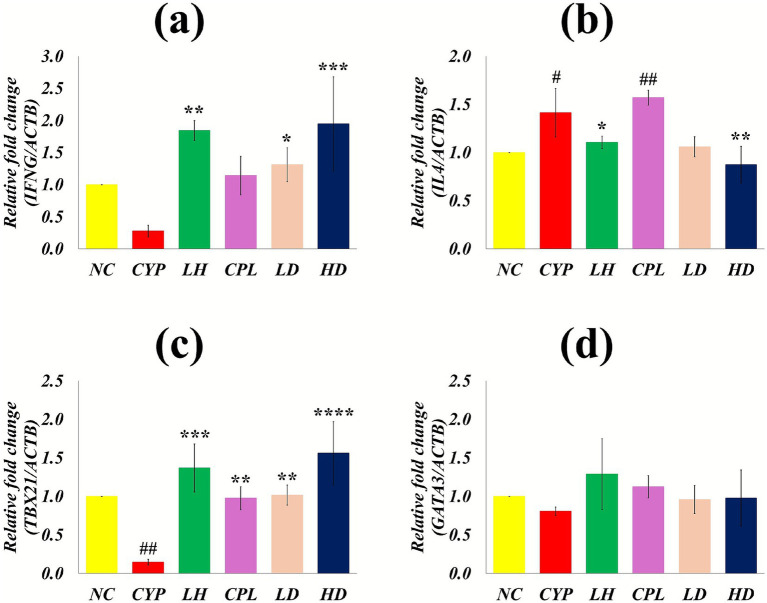
Relative fold change in Th1 and Th2 marker gene expression after SCPH treatment. **(A)** *IFNG* **(B)** *IL4* **(C)** *TBX21* **(D)** *GATA3* mRNA expression. Results are shown with mean ± SD (*n* = 4). ^#^*p* < 0.05, ^##^*p* < 0.005, ^###^*p* < 0.0005, ^####^*p* < 0.0001 compared with NC group; ^*^p < 0.05 ^**^p < 0.005, ^***^p < 0.0005, ^****^p < 0.0001 compared with CYP group.

Gene expression and relative quantification of Th1-associated (*TBX21, IFNG*) and Th2-associated (*GATA3, IL4*) genes were performed by two-step RT–qPCR. This transcriptional profiling was used to assess restoration of immune homeostasis in SCPH-treated mice following CYP-induced immunosuppression. *IFNG* and *TBX21* mRNA levels were significantly reduced in the CYP group compared with the NC group (*p* > 0.05 and *p* = 0.006, respectively), whereas SCPH treatment markedly increased their splenic expression, consistent with reactivation of Th1 responses. In contrast, IL4 mRNA expression was significantly elevated in the CYP (*p* = 0.034) and CPL (*p* = 0.004) groups relative to NC but was significantly lower in the SCPH high-dose group, indicating attenuation of the CYP-induced Th2 shift. *GATA3* expression showed a trend toward upregulation in the CYP and CPL groups compared with NC; SCPH did not significantly change *GATA3* levels but showed an apparent trend toward downregulation relative to CYP, as illustrated in Figures 5A–D, consistent with the reciprocal regulation of *TBX21* and *GATA3* in Th1/Th2 balance.

### 3.4 SCPH rebalances the Th1/Th2 serum cytokines and immunoglobulin production

Serum levels of Th1-associated cytokines (IFNG, IL1B) and Th2-associated cytokines (IL4, IL10) were quantified using highly sensitive double-antibody sandwich ELISAs, and IgG, IgM, and IgA concentrations were measured by inverse antigen competitive ELISA. CYP treatment significantly suppressed IFNG and IL1B in serum (CYP: *p* = 0.006 and *p* = 0.001; CPL: *p* = 0.048 and *p* = 0.028 vs. NC), whereas SCPH administration restored these cytokines toward normal levels, consistent with recovery of Th1-mediated responses. In contrast, IL4 and IL10 were elevated in the CYP (*p* = 0.048 and *p* > 0.05) and CPL groups relative to NC, and high-dose SCPH significantly reversed this Th2-skewing, normalizing Th2 cytokine levels.

**Figure 6:**
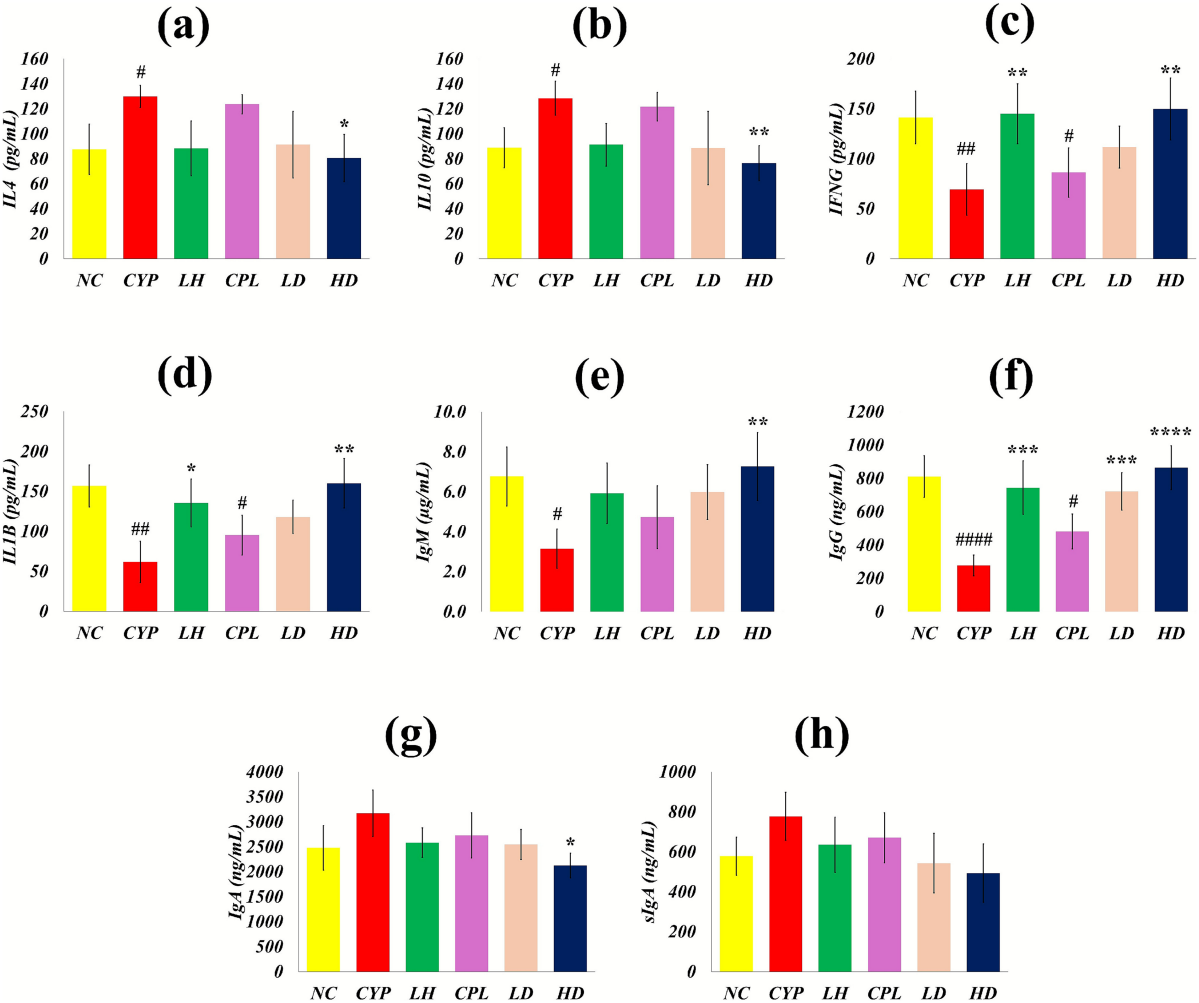
Estimation of Th1 and Th2 cytokines and immunoglobulins after SCPH treatment. Results are shown with Mean ± SD (*n* = 4). ^#^*p* < 0.05, ^##^*p* < 0.005, ^###^*p* < 0.0005, ^####^*p* < 0.0001 compared with NC group; ^*^p < 0.05 ^**^p < 0.005, ^***^p < 0.0005, ^****^p < 0.0001 compared with CYP group.

Humoral markers mirrored these cytokine changes. Serum IgG and IgM were significantly reduced in the CYP group (*p* < 0.0001 and *p* = 0.025 vs. NC), paralleling the decline in Th1 cytokines and indicating impaired class-switched and early antibody responses, while SCPH treatment increased IgG and IgM, suggesting recovery of B cell function and T cell help. By contrast, IgA and sIgA were elevated in CYP-treated mice, consistent with a Th2-driven and mucosally biased response; this pattern reflects a shift toward Th2 polarization and enhanced mucosal-type immunity despite systemic immunosuppression. Collectively, these data indicate that CYP represses Th1-associated cytokines and IgG/IgM while favoring Th2-associated cytokines and IgA/sIgA, and that SCPH treatment rebalances these serum markers toward a more physiological Th1/Th2 equilibrium (Figures 6A–H).

### 3.5 SCPH ameliorates histomorphology features of spleen tissue

**Figure 7:**
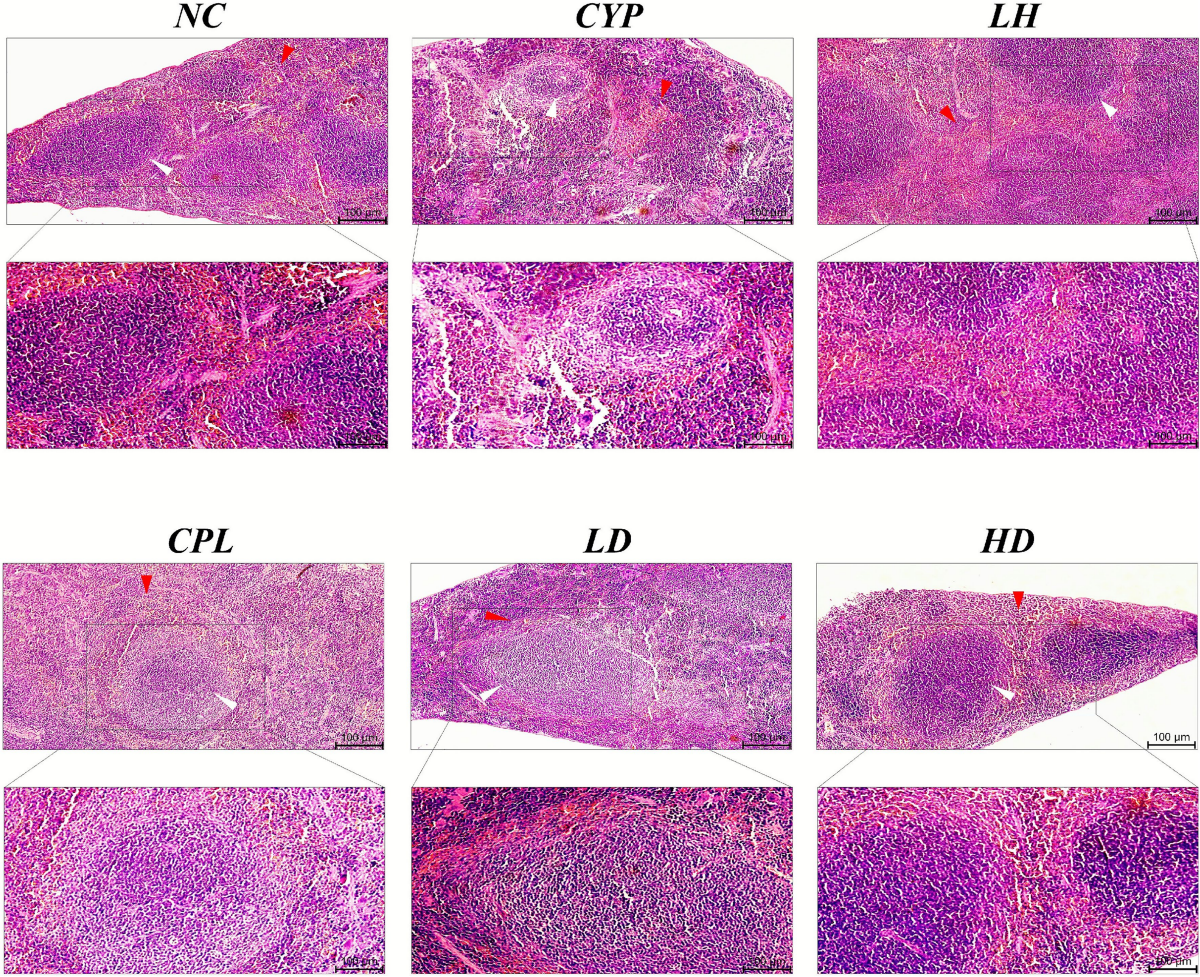
Effects of SCPH on spleen histology by HE staining; red arrowheads illustrate red pulp (large number of red blood cells) region, white arrowheads show white pulp regions (nuclei-rich follicular marks), white arrows depict the distribution of PALS, and black arrows indicate marginal zones between red and white pulp, in splenic histology. Magnification parameters: upper 10×, lower inset 20×, scale bar = 100 μm.

The histological visualization of the spleen reveals the prominent changes in spleen tissue morphology. The NC and SCPH-treated groups have shown opulent, well-defined, and dense lymphoid morphology in white pulp regions. The marginal zones were well demarcated. The red pulp shows healthy cords of Billroth (irregular cellular cords) and splenic sinusoids (clear, elongated spaces surrounding the darker white pulp). Conversely, CYP reduces the presence of lymphocyte density, depleting the follicular B cells and T cells in the periarteriolar lymphoid sheaths (PALS) of the white pulp. CYP apparently disrupts the marginal zones between red and white pulp. The chronic damage seen in the cords and sinusoids of the red pulp is due to immune dysfunction in CYP. The CPL group represents similar features to CYP as seen in Figure 7.

### 3.6 SCPH equilibrizes T-bet/GATA3 histochemical protein expression in spleen

Immunohistochemical staining of spleen sections for the T-bet and GATA3 proteins was used to assess their spatial distribution within the splenic architecture, reflecting their roles in immunoregulation and T helper cell differentiation. DAB-based detection revealed markedly reduced T-bet expression in CYP, CPL, and SCPH high-dose (HD) treated groups compared with NC (CYP: *p* = 0.0001; CPL: *p* = 0.003; HD: *p* < 0.0001), indicative of impaired Th1 differentiation and weakened type 1 immunity against intracellular pathogens. In contrast, SCPH treatment produced a dose-dependent restoration of T-bet, with significantly increased expression in the low-dose (LD; *p* < 0.0004) and HD (*p* < 0.0001) groups versus CYP, consistent with partial recovery of Th1-associated transcriptional programming.

**Figure 8:**
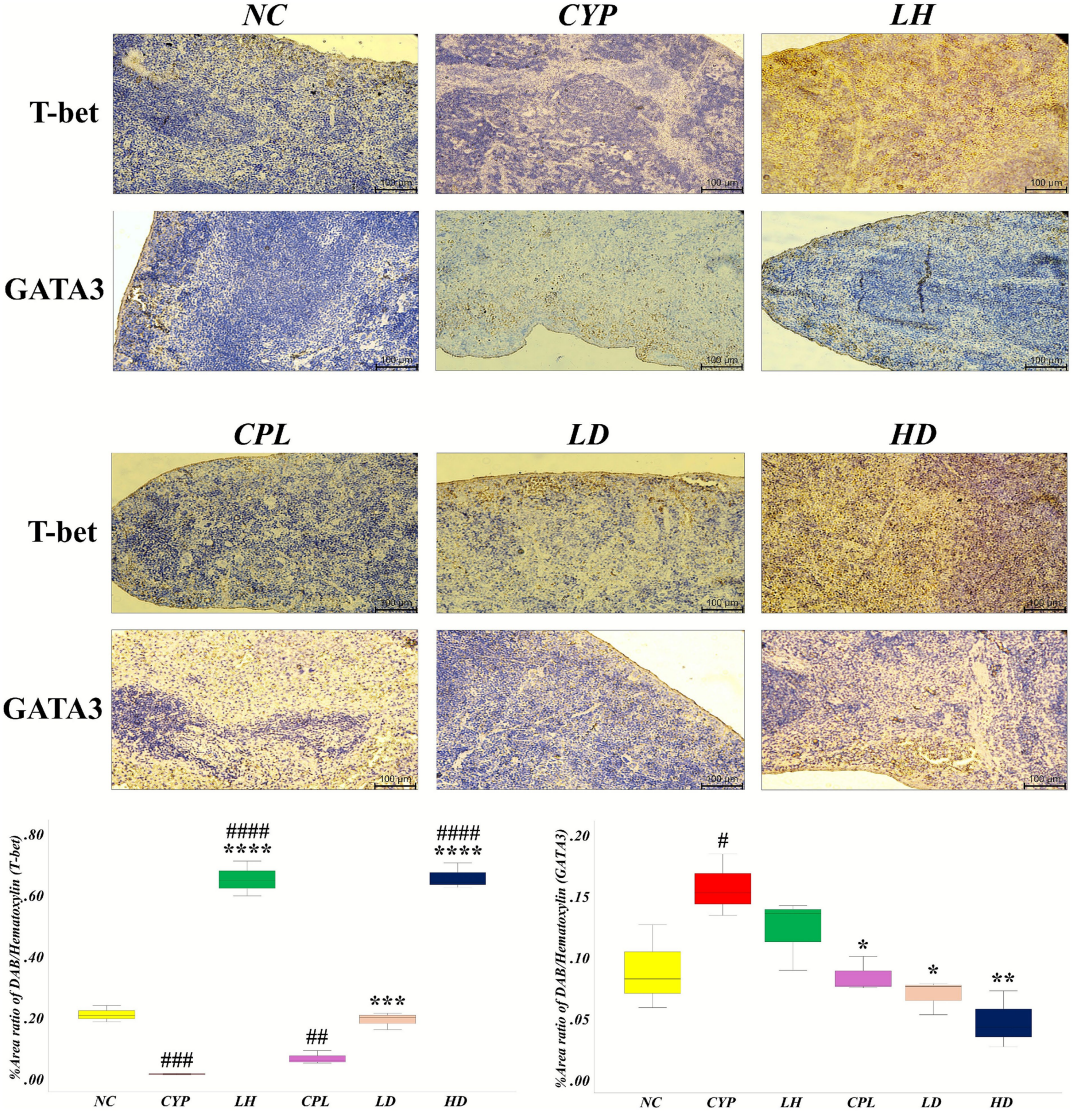
Modulatory effects of SCPH on splenic expression of T-bet and GATA3 protein (brownish), and nuclei (blue) by immunohistochemistry (IHC); magnification parameters: upper 10×, lower inset 20×, scale bar = 100 μm. Results are shown with mean ± SD (*n* = 4). ^#^*p* < 0.05, ^##^*p* < 0.005, ^###^*p* < 0.0005, ^####^*p* < 0.0001 compared with NC group; ^*^p < 0.05 ^**^p < 0.005, ^***^p < 0.0005, ^****^p < 0.0001 compared with CYP group.

GATA3 staining showed relatively higher expression in the CYP group compared with NC (*p* = 0.046), consistent with a shift toward Th2 polarization and enhanced humoral-type responses. SCPH treatment reduced GATA3 levels, with significantly lower expression in the LD (*p* = 0.009) and HD (*p* = 0.001) groups versus CYP, suggesting attenuation of CYP-induced Th2 skewing. Together, these changes in T-bet and GATA3 protein expression support a SCPH-mediated rebalancing of Th1/Th2 regulation in the spleen (Figure 8).

## 4 Discussion

The marine protein hydrolysates absorb vigorously rather than the intact proteins; due to this bioavailability, they ameliorate nutrient absorption and utilization. Bioactively, *A. japonicus* is the richest source of collagen type-I among other aquatic sources (14, 19). *Clostridium-derived* collagenases are widely used for collagen hydrolysis because they are safe, efficient, and highly reproducible, generating peptides that retain the nutritional and functional properties of the parent protein. Enzymatic hydrolysis improves the nutritional quality and therapeutic potential of functional foods, typically producing low–molecular–weight peptides of approximately 2–20 residues, whose bioactivity is influenced by sequence length, with shorter peptides being more readily absorbed and longer peptides often exhibiting greater stability and functional persistence in the intestinal mucosa (20–22).

Peptides enriched in glycine, glutamine, and asparagine display notable immunomodulatory and anti-inflammatory properties, particularly on innate immune cells such as monocytes and macrophages. These peptides can modulate cytokine secretion, regulate immunoglobulin production, and protect tissues from oxidative stress, thereby contributing to systemic immune support (23–25). In LC–MS/MS analysis, the precursor m/z denotes the mass-to-charge ratio of the peptide ion selected for fragmentation and depends on the ion charge state, while the retention time corresponds to the chromatographic elution time used to resolve peptides before MS detection. The X-Corr (cross-correlation) score reflects the match quality between observed and theoretical MS/MS spectra, with higher scores indicating greater confidence, and the assigned charge state (commonly +2 or +3) influences both m/z and fragmentation patterns; together, these parameters support accurate peptide identification in proteomics workflows (26–28). In BLASTp searches against non-redundant protein databases, the maximum and total alignment scores describe local and cumulative alignment quality, respectively, while low E-values and high percent identity indicate statistically significant and biologically meaningful sequence similarity, helping to validate peptide or protein annotation. Based on prior work, SCPH contains high-quality peptides that can be further purified or synthesized for potential clinical applications (29–31).

Balanced Th1/Th2 immune homeostasis is essential for effective host defense while preventing excessive inflammation and immunopathology. Th1 responses, characterized by IFNG, IL1B, and TNFA production, drive cell-mediated immunity against intracellular pathogens and tumor cells but can promote autoimmunity when overactivated. In contrast, Th2 responses, driven by IL4, IL5, IL10, and IL13, support humoral immunity against extracellular pathogens and promote IgE production in allergic disease; their overactivation is associated with allergy and asthma (32–34).

Spleen and thymus are the critical biomarkers of the immune system, and their atrophy ensures eminent immunosuppression. Previous studies suggest that CYP induces apoptosis in the rapidly dividing immune cells, ultimately causing the shrinkage of spleen, thymus, and lymph nodes. Reduced splenic index illustrates compromised peripheral immune responses, and thymic index reflects impaired T-cell maturation, while a reduction in mesenteric lymph node index correlates with impaired gut-associated lymphoid tissue (GALT) functioning and intestinal pathogens (35–37). Consistent with earlier reports, the present findings show that SCPH treatment counteracts CYP-induced organ atrophy, supporting the regrowth and functional recovery of major immune organs.

Cyclophosphamide induces profound leukopenia by suppressing bone marrow hematopoiesis, with lymphopenia reflecting its selective toxicity toward rapidly dividing T and B lymphocytes and thus impaired adaptive immunity. Monocytes and granulocytes, key first-line effector cells, also exhibit CYP-mediated alterations; increased percentages of these subsets often represent relative shifts caused by a marked fall in total WBC and neutrophil counts rather than true expansion. In this context, the proportional changes seen in the CYP and SCPH high-dose groups likely reflect compensatory proliferation and mobilization of immune cells in response to tissue damage, with groups showing reduced abnormal percentages indicating normalization of leukocyte composition as total WBC counts recover (38, 39).

T-bet (*TBX21*) and *GATA3* function as mutually antagonistic master regulators of Th1 and Th2 differentiation, respectively, establishing transcriptional programs that favor expression of lineage-specific cytokines while repressing alternative fates. T-bet can bind directly to GATA3 and alter its DNA-binding profile, thereby preventing activation of Th2 genes while promoting Th1 gene expression, whereas IL4 signaling enhances GATA3 expression and IFNG supports sustained *TBX21* expression, together determining Th1/Th2 balance. Disruption of this axis, including combined loss of T-bet and GATA3, results in severe autoimmune-like pathology due to loss of appropriate immune polarization and homeostasis (22, 39). In line with previous studies reporting elevated IL4, GATA3, and IL10 expression under immunosuppressive conditions and their reduction following effective intervention, SCPH treatment in this study restored TBX21 and GATA3 mRNA expression patterns compatible with a more balanced Th1/Th2 profile.

Reduced IFNG, IL1B, IgG, and IgM levels in CYP-induced immunosuppression indicate compromised B-cell function and antibody production, likely due to direct B-cell cytotoxicity and defective T-cell help. Conversely, increases in these Th1-associated cytokines and immunoglobulins enhance phagocyte and NK-cell activity, facilitate pathogen clearance, and rescue antibody production by supporting B-cell proliferation and differentiation (18, 40). In the present work, SCPH administration increased IFNG, IL1B, IgG, and IgM, consistent with Th1 restoration; IFNG drives IgG subclass switching and indirectly promotes IgM responses by improving antigen presentation to B cells, while IL1B, preferentially induced in Th1-skewed environments, amplifies proinflammatory T cell responses and supports the expansion of IFNG-producing effector subsets. IL4 and IL10 are important inducers of IgA synthesis, enhancing plasma cell differentiation and expansion of IgA-producing B cells (41–43), so elevated IgA under conditions of overall immunosuppression may reflect a compensatory mucosal immune response to CYP-induced intestinal barrier perturbation. Similarly, the parallel increase in sIgA, together with IL4, IL10, and IgA, suggests that SCPH not only restores systemic humoral immunity but also reinforces mucosal barrier defense, as sIgA is the predominant antibody at mucosal surfaces and is critical for immune exclusion of luminal pathogens and maintenance of intestinal homeostasis. These coordinated changes in Th2-associated cytokines, IgA, and sIgA therefore indicate that SCPH promotes integrated systemic and mucosal immune recovery in the CYP-immunosuppressed state (44).

The white pulp is composed of lymphoid tissue, and is responsible for T and B cell activation, antibody production, and defense against encapsulated bacteria and organized into three compartments; (a) the periarteriolar lymphoid sheath (PALS), which surrounds the central artery and rich in T lymphocytes, (b) lymphoid follicles, contains B lymphocytes and site of antibody production, (c) marginal zone, located between white and red pulp which contains B cells and antigen-presenting cells. The red pulp makes up 76–79% of a normal spleen and consists of: (a) cords of Billroth, a network of connective tissue containing macrophages, plasmocytes, and blood cells, (b) splenic sinusoids consist of wide blood vessels that filter blood and remove old or damaged red blood cells (45).

The spleen’s white pulp, composed of periarteriolar lymphoid sheaths (T cell–rich), lymphoid follicles (B cell–rich), and the marginal zone (B cells and antigen-presenting cells), orchestrates adaptive immune activation, whereas the red pulp, comprising cords of Billroth and splenic sinusoids, filters blood and removes senescent erythrocytes. Cyclophosphamide treatment is associated with loss of clear demarcation between red and white pulp, depletion of lymphocytes, reduced germinal centers, and architectural disorganization, changes that correlate with impaired immune activation and increased susceptibility to infection (46–48). Red pulp atrophy reduced hematopoietic precursors, and increased IL10 further exacerbate immunosuppression and structural deterioration, including blurred red–white pulp boundaries and damaged marginal zones (49–51). In consonance with these histopathological patterns, CYP and CPL-treated spleens exhibited white and red pulp deterioration, whereas SCPH-treated spleens showed restoration of lymphoid architecture, lymphocyte repopulation, and germinal center reactivation, supporting renewed antibody production.

Suppressed T-bet and enhanced GATA3 expression in immunosuppressed spleens reflect the shift toward a Th2-polarized state driven by elevated IL4 and reduced IFNG, which favor GATA3 induction and limit T-bet expression (52, 53). The immunohistochemical data showing decreased T-bet and increased GATA3 protein in CYP-treated splenic tissue provide protein-level evidence of this regulatory imbalance. SCPH treatment normalized T-bet and GATA3 expression, indicating recovery of Th1/Th2 master regulator activity and counteracting CYP-induced immune dysregulation at both transcriptional and protein levels. We acknowledge that the present study is primarily descriptive and focuses on system-level immunological outcomes, including cytokine profiles, immunoglobulin production, transcription factor expression, and histopathological changes. Accordingly, this work should be considered a proof-of-concept *in vivo* investigation demonstrating the immunomodulatory potential of SCPH rather than a comprehensive mechanistic dissection. While the observed immunological effects are robust, the precise cellular and molecular mechanisms underlying SCPH action were not fully elucidated in this study. Future investigations will aim to address these limitations by performing *in vitro* T-cell differentiation and polarization assays in the presence of SCPH, fractionating bioactive peptides to identify functional components, and interrogating relevant receptor-mediated pathways, such as Toll-like receptors and metabolite-sensing G protein–coupled receptors. In addition, the use of knockout or reporter mouse models will be valuable for dissecting pathway-specific involvement in SCPH-mediated immune regulation. Given the close interplay between dietary peptides and host–microbiota interactions, comprehensive gut microbiota analyses will also be pursued to determine whether microbial modulation contributes to the observed immunological effects. Together, these future studies will build upon the current findings and provide mechanistic depth to the immunomodulatory actions of SCPH.

## 5 Conclusion

SCPH mitigated CYP-induced immunosuppression, improving body weight and immune organ indices, normalizing selected immunoglobulins, and favorably modulating Th1-/Th2-related cytokines and transcription factors, alongside histological protection. CPL produced partial immunoregulatory effects, indicating that intact or less-hydrolyzed proteins retain bioactivity, but SCPH exerted broader and more consistent actions on Th1-associated markers, suggesting added benefit of peptide enrichment while acknowledging that processing differences and the absence of alternative hydrolysates or prebiotics limit attribution of specificity. The cytokine pattern is best described as a Th1-oriented restoration under CYP-induced suppression rather than a symmetric re-balancing, which may favor anti-tumor or anti-infective responses but warrants future mechanistic work, including T-cell differentiation assays, flow cytometry of Th subsets, receptor-pathway studies and gut-mucosal investigations, to define cellular targets, long-term safety and the contribution of mucosal immunity.

## Data Availability

The raw data supporting the conclusions of this article will be made available by the authors upon reasonable request.
